# Effect of bulky anions on the liquid-liquid phase transition in phosphonium ionic liquids: Ambient and high-pressure dielectric studies

**DOI:** 10.1038/s41598-023-29518-8

**Published:** 2023-02-21

**Authors:** B. Yao, M. Paluch, Z. Wojnarowska

**Affiliations:** Institute of Physics, University of Silesia, SMCEBI, 75 Pulku Piechoty 1A, 41-500 Chorzow, Poland

**Keywords:** Phase transitions and critical phenomena, Structure of solids and liquids, Fluid dynamics

## Abstract

Although the first-order liquid–liquid phase transition (LLT) has been reported to exist in various systems (i.e., phosphorus, silicon, water, triphenyl phosphite, etc.), it is still one of the most challenging problems in the field of physical science. Recently, we found that this phenomenon occurs in the family of trihexyl(tetradecyl)phosphonium [P_666,14_]^+^ based ionic liquids (ILs) with different anions (Wojnarowska et al in Nat Commun 13:1342, 2022). To understand the molecular structure–property relationships governing LLT, herein, we examine ion dynamics of two other quaternary phosphonium ILs containing long alkyl chains in cation and anion. We found that IL with the anion containing branched –O–(CH_2_)_5_–CH_3_ side chains does not reveal any signs of LLT, while IL with shorter alkyl chains in the anion brings a hidden LLT, i.e., it overlaps with the liquid-glass transition. Ambient pressure dielectric and viscosity measurements revealed a peculiar behavior of ion dynamics near T_g_ for IL with hidden LLT. Moreover, high-pressure studies have shown that IL with hidden LLT has relatively strong pressure sensitivity compared to the one without first-order phase transition. At the same time, the former exposes the inflection point indicating the concave-convex character of logτ_σ_(P) dependences.

## Introduction

In the past decades, the interest in ionic liquids (ILs), fluids composed solely of ions, has risen tremendously, especially in the fields of chemistry, materials science, and energy research^1^. This is because a change of cation or anion can significantly tune the physicochemical properties of IL. Good chemical and thermal stability, high ionic conductivity (> 10^−4^ S cm^−1^), negligible vapor pressure, and non-flammability make ILs promising electrolyte^2^ and green solvents for applications in electrochemistry^3^, biology, and pharmacology^4,5^.

Compared to cyclic ILs containing imidazolium, pyrrolidinium, or piperidinium cations, quaternary phosphonium (P) ILs have many valuable physicochemical properties, e.g., enhanced hydrophobicity and significantly wider electrochemical window^6^. Furthermore, they exhibit relatively low viscosity and thus enhanced ionic conductivity at RT conditions^7,8^. The origin of these essential features has been examined over the years by several groups. Among the molecular factors which may contribute to the lower viscosity of phosphonium ILs the size of the phosphorous atom, the charge distribution, and the flexibility of bond and dihedral angles were examined. Furthermore, a large volume of phosphonium cations that result from the shielding of phosphonium core by charge-neutral alkyl groups and bring lower electrostatic friction between counterions was also considered^9,10^.

Extensive investigations of charge transport in phosphonium ionic liquids have shown that the dc conductivity (*σ*_dc_) is closely coupled to their structural (*α*) relaxation^11^, which is related to the viscosity (*η*) by the Maxwell equation, *η* = *G*_∞_/*τ*_α_^12^. Thus, the classical vehicle mechanism characterizes the ion transport in phosphonium ILs. Furthermore, these materials have been reported in the literature as good glass-formers, i.e., they can form an amorphous solid if sufficiently fast cooling is applied^13^. Over a relatively narrow temperature range, from RT to the temperature of liquid-glass transition (*T*_g_), the viscosity of P-ILs increases dramatically, even by ten orders of magnitude. At the same time, σ_dc_ decreases in the same manner, commonly described by a single Vogel-Fulcher-Tamman (VFT) equation ${\sigma }_{dc}={\sigma }_{0}exp(D{T}_{0}/(T-{T}_{0}))$, where σ_0_ is a pre-exponential factor, and D quantifies the departure of experimental data from the Arrhenius law. An alternative measure of the non-Arrhenius conductivity and fluidity behavior is the fragility index m_P_ defining the steepness of the temperature dependence of various transport properties at *T*_g_. The phosphonium ionic liquids studied so far fall into the intermediate range of fragilities (*m*_P_ = 46–60) compared to other ionic liquids and glassformers in general^14^.

Such a well-defined physical picture of ion dynamics of P-RTILs has been upended by recent studies of trihexyl(tetradecyl)phosphonium [P_666,14_]^+^ based ionic liquids. In particular, it has been shown that at a certain temperature, dc-conductivity and conductivity relaxation times *τ*_σ_ = *ε*_0_*ε*_s_*σ*_dc_^−1^, reveal a substantial departure from VFT law accompanied by an abrupt increase in apparent activation energy^15,16^ (note that during the liquid-glass transition, an apparent activation energy decreases^17^). This peculiar behavior, reported for five different [P_666,14_]^+^-based ILs, has been recognized as the dielectric signature of the first-order liquid–liquid phase transition (LLT). Strictly speaking, two isotropic liquid states of different dc-conductivity behavior were found to exist within a single IL. As a molecular origin of this unique phenomenon, enhanced ordering of cation alkyl chains in non-polar domains was postulated from XRD and IR experiments^16^.Furthermore, it has been shown that LLT occurs at elevated pressure. Namely, isothermal compression triggers the LLT, with the *τ*_σ_ at LLT being independent of temperature and pressure conditions. The time scale of ion dynamics at LLT was found anion-specific, i.e., dependent on anion size, geometry, conformational flexibility, and the strength of interionic interactions^15^. Furthermore, it was postulated that large anions most likely complicate the ordering of the alkyl chains in the non-polar domains and consequently prevent the LLT^15^.

To further advance the issue of liquid–liquid phase transition in ionic systems, herein we focus on ion dynamics of two ILs containing [P_666,14_]^+^ cation and phosphorus-based anions with long alkyl chains as well. The results of dielectric experiments performed over a wide temperature range revealed that both examined ILs display the conventional dc-conductivity behavior without a clear sign of LLT. However, a more detailed analysis of experimental data indicated that one of the chosen ILs exhibits a hidden LLT. High-pressure dielectric experiments performed for both ILs, have shown distinct differences in their pressure sensitivity of ion dynamics. Furthermore, another unique phenomenon called “inflection point” has been observed for one of examined herein ILs.

## Results and discussion

**Figure 1:**
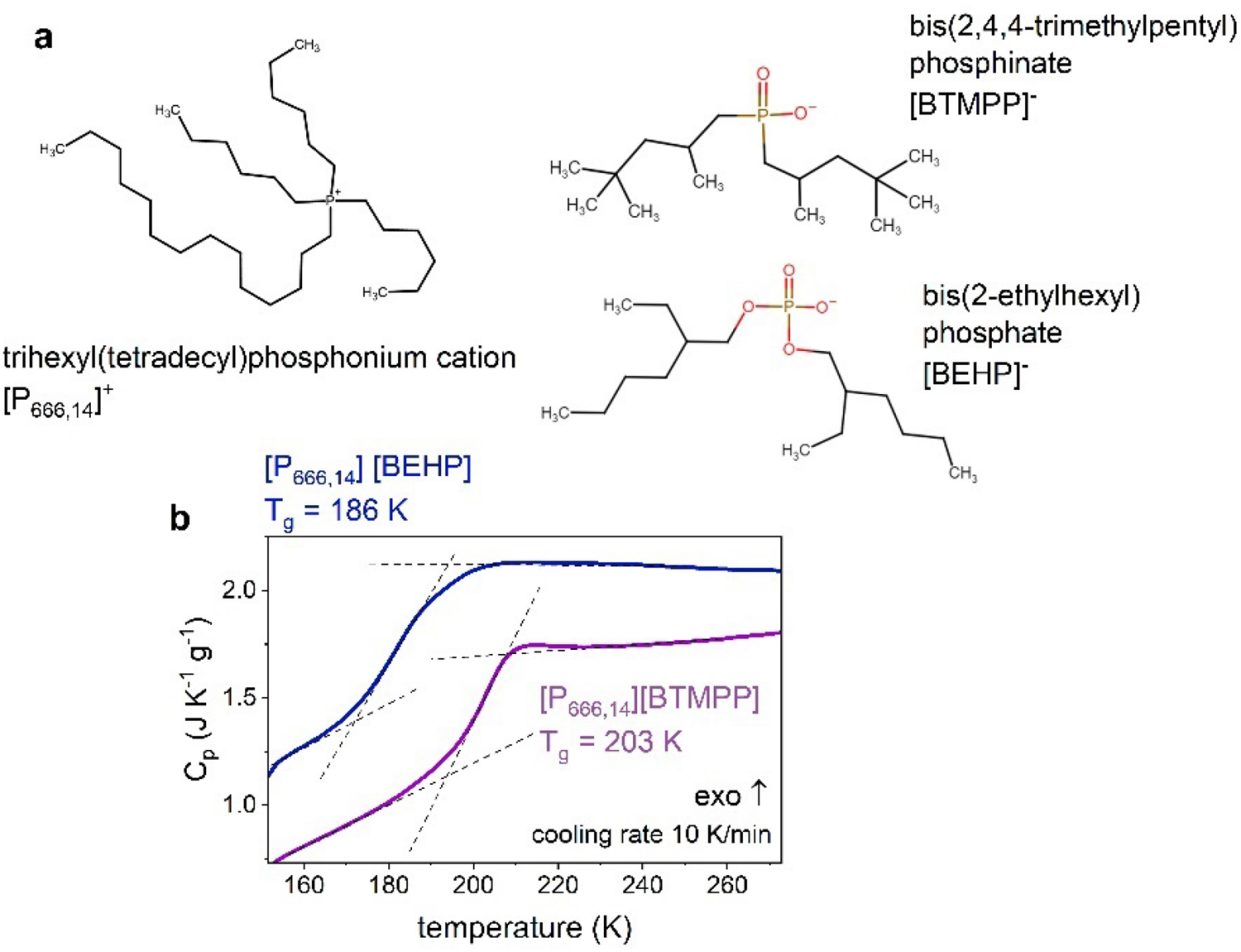
Initial characterization of studied ILs. (**a**) Chemical structure of trihexyl(tetradecyl)phosphonium cation [P_666,14_]^+^ and anions: bis(2-ethylhexyl) phosphate [BEHP]^-^, bis(2,4,4-trimethylpentyl) phosphinate [BTMPP]^−^. (**b**) Differential scanning calorimetry (DSC) traces of [P_666,14_][BEHP] and [P_666,14_][BTMPP] obtained on cooling with the rate of 10 Kmin^−1^.

The ILs examined in this work are two quaternary phosphonium ionic liquids with hydrophobic [P_666,14_]^+^ cation and anions: bis(2-ethylhexyl) phosphate ([BEHP]^-^) and bis(2,4,4-trimethylpentyl) phosphinate ([BTMPP]^−^). The chemical structures are displayed in Fig. 1a. In analogy to cation, the anions reveal non-cyclic nature with two long and branched alkyl chains connected to a phosphorus core. Specifically, in the [BEHP]^-^ structure, two six-carbon chains are attached to phosphorus through the oxygen atom. On the other hand, five carbon chains characterize the [BTMPP]^-^ anion. The van der Waals volume of anions, determined by the Bondi method^18^, is equal to ${V}_{[BEHP]}^{vdW}$=0.339 nm^3^ and ${V}_{[BTMPP]}^{vdW}$=0.321 nm^3^. Consequently, the former provides more sterical hindrance that might affect the alignment of the nonpolar alkyl chains of [P_666,14_] cation, suggested as the origin of the liquid–liquid transition. Moreover, oxygen atoms in [BEHP]^−^ make the side chains polar, which can also influence the LLT.

Differential scanning calorimetry (DSC) measurements have been performed for initial characterization of [P_666,14_][BTMPP] and [P_666,14_][BEHP]. Importantly, before the experiments, both examined samples were extensively dried at 343 K under vacuum for 12 h. The DSC thermograms obtained at a cooling rate of 10 Kmin^−1^ of examined ILs are given in Fig. 1b. It can be seen that both ILs exhibit a step-like increase in heat capacity during the cooling process, which is a typical manifestation of the liquid-glass transition. However, on the heating scan, there are also some unidentified endothermic effects of small intensity (see Fig. S1). Since they are not observed on the cooling trace, one can exclude the LLT as the origin of these thermal events. At the same time, one can suppose that the other peaks visible on the heating scan are due to the crystallization of impurities in examined samples. Thus, at this point, the calorimetric results only indicated the occurrence of the typical glass transition in examined ILs and did not provide any signs of the first-order liquid–liquid transition.

**Figure 2:**
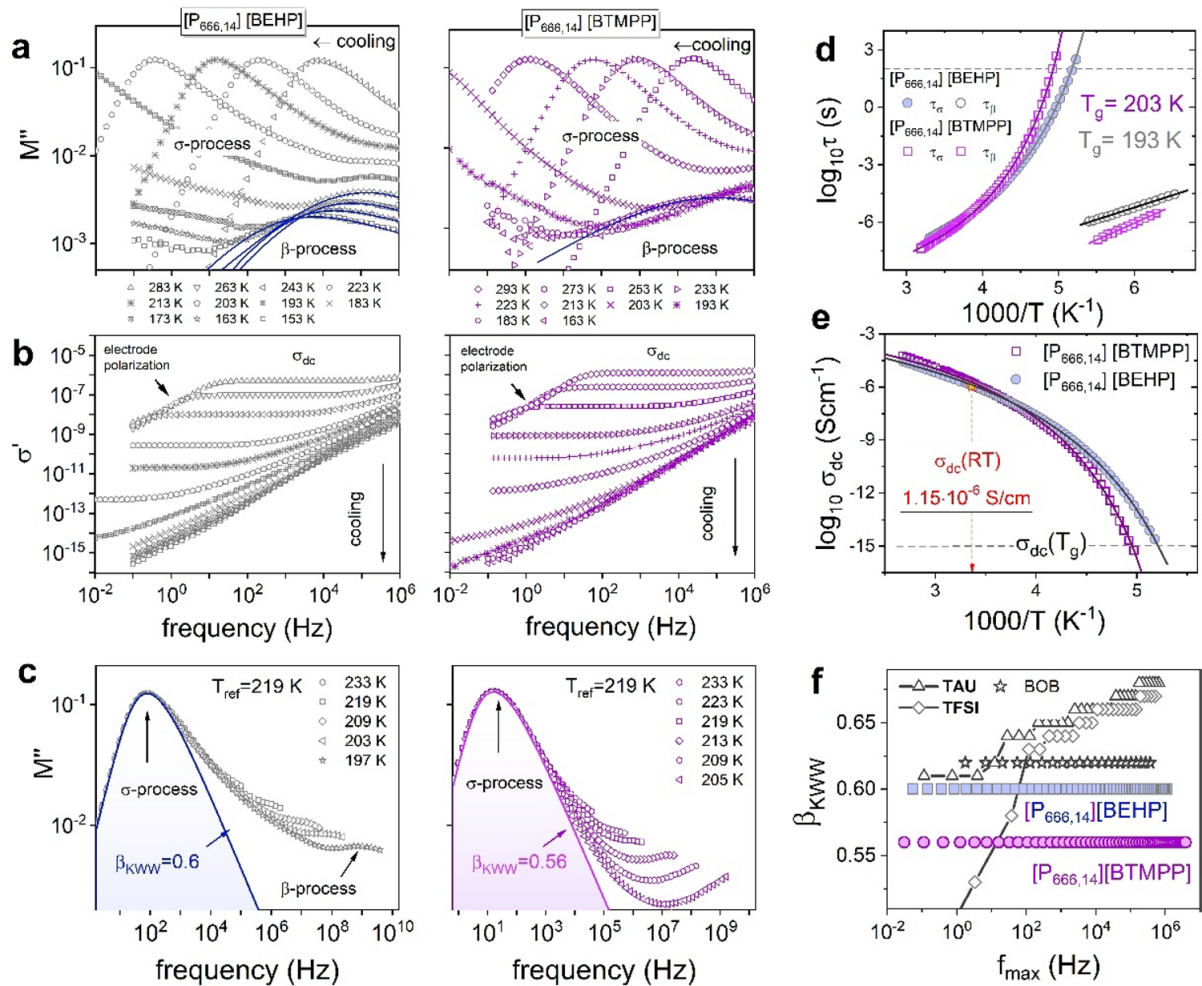
Dielectric response of examined ILs measured at ambient pressure. (**a**) Imaginary part of the complex electric modulus *M*’’ and (**b**) real part of complex conductivity *σ*’ as a function of frequency at various temperatures for [P_666,14_][BEHP] and [P_666,14_][BTMPP]. (**c**) Superimposition of *M*’’ spectra of [P_666,14_][BEHP], [P_666,14_][BTMPP] at 0.1 MPa and several temperatures. (**d**) Temperature dependence of conductivity relaxation times above and below *T*_*g*_. (**e**) Temperature dependence of dc-conductivity. Solid lines in (**d**) and (**e**) denote the fits of the VFT equation to experimental data above *T*_*g*_ and the fits of Arrhenius law for the secondary relaxation process below *T*_*g*_. Adj. R-Square of VFT fits is equal to 0.9999 and 0.9992 for [P_666,14_][BEHP], [P_666,14_][BTMPP], respectively. (**f**) *β*_KWW_ as a function of *M*”(f) peak maximum for [P_666,14_][BEHP], [P_666,14_][BTMPP] and three others IL, i.e. [P_666,14_][BOB], [P_666,14_][TAU] and [P_666,14_][TFSI].

To characterize the ion dynamics of [P_666,14_][BTMPP] and [P_666,14_][BEHP] ILs through the liquid-glass transition, temperature-dependent dielectric experiments have been performed. Herein, we employed two formalisms, complex electric modulus M^*^(*f*) and complex conductivity σ^*^(*f*), to present the obtained data. Both of them are commonly adopted to express the charge transport properties of conducting materials^19–21^. The representative dielectric spectra of [P_666,14_][BEHP] and [P_666,14_][BTMPP] measured over eight decades of frequency (10^−2^–10^6^ Hz) in a wide temperature range are illustrated in Fig. 2a,b, in modulus and conductivity formalism, respectively. In both examined cases, the imaginary part of the complex modulus *M*”(*f*) recorded in a supercooled liquid state takes the form of a well-resolved peak, so-called a conductivity relaxation (denoted as *σ*-process), which shifts toward lower frequencies upon cooling. This behavior reflects the suppressed mobility of ions and is in keeping with the cooling effects revealed in all kinds of ionic glass formers^22^. Further decreasing temperature below the calorimetric *T*_*g*_ brings the modulus peak out of the experimental window, and a well-distinguished secondary mode (*β*) becomes visible. Similar to the conductivity relaxation, the *β*-process move toward lower frequencies on cooling; however it is far less temperature-dependent. At the same time, the real part of complex conductivity *σ*’(*f*) is characterized by three clearly visible regions: (i) a frequency-independent dc-conductivity (*σ*_dc_) that is proportional to the number of ions and their mobility, (ii) a power-law behavior obeyed on the high-frequency side, (iii) a decrease of *σ*’ from *σ*_dc_ denoting the electrode polarization effect. Note that both formalisms, *M*”(*f*) and *σ*’(*f*), adopted herein for data analysis, provide information on the ion mobility in a supercooled liquid state; however, compared to modulus representation, dc-plateau is well visible above room temperature. Therefore, the temperature dependence of *σ*_dc_ over 11 orders of magnitude can be examined. At the same time, only the modulus representation offers a characterization of dynamics in a glassy state.

Taking the last mentioned advantage, we have examined the dynamics of glassy [P_666,14_][BEHP] and [P_666,14_][BTMPP] in more detail. For this purpose, a numerical fitting analysis of *M*”(*f*) data, in terms of the Havriliak−Negami function (Eq. 1)^23^, has to be carried out.

1 $$M^{*} \left( \omega \right) = 1/\varepsilon^{*} \left( \omega \right) = \left( {\varepsilon_{\infty } + \frac{\Delta \varepsilon }{{[1 + (i\omega \tau_{HN} )^{{\alpha_{HN} }} ]^{{\beta_{HN} }} }}} \right)^{ - 1}$$

where Δ*ε* is the dielectric strength, *ε*_*∞*_ denotes the high-frequency limit permittivity, *τ*_*HN*_ is the characteristic relaxation time and the exponents *α*_*HN*_ and *β*_*HN*_ represent the symmetric and asymmetric broadenings of the dielectric loss curve. Having the values of fitting parameters, we calculated the characteristic relaxation times of *β*-mode observed in modulus spectra below *T*_g_:

2 $$\tau_{\beta } = \tau_{HN} \left[ {\sin \left( {\frac{{\alpha_{HN} \cdot \pi }}{{2 + 2\beta_{HN} }}} \right)} \right]^{{ - 1/\alpha_{HN} }} \left[ {\sin \left( {\frac{{\alpha_{HN} \cdot \beta_{HN} \cdot \pi }}{{2 + 2\beta_{HN} }}} \right)} \right]^{{1/\alpha_{HN} }}$$

The obtained temperature variations in *τ*_*β*_ are depicted in Fig. 2d. As can be seen, log*τ*_*β*_(*T*^−1^) data of both examined ILs obeys the Arrhenius law ${\tau }_{\beta }={\tau }_{\infty }exp(\frac{{E}_{a}}{RT})$ with the energy barrier equal to 24.4 ± 0.2 kJ/mol and 33.9 ± 0.5 kJ/mol, for [P_666,14_][BEHP] and [P_666,14_][BTMPP], respectively. Considering the relatively low activation energy of these modes, one can assume their intramolecular origin—specifically, rotations of anion or cation alkyl chains. The higher E_a_ determined for relaxation of 5-carbon anion’s chains can be rationalized by more extended branching compared to the side groups of [BEHP]^-^ anion. The coupling model (CM) predictions have been performed to confirm the intramolecular origin of secondary modes. As can be seen in Fig. S2, the temperature dependence of *τ*_*JG*_ (denoting the potential position of a secondary mode of intermolecular origin) for [P_666,14_][BTMPP] and [P_666,14_][BEHP] does not match with the relaxation times of the *β*-process, indicating their intramolecular nature.

To describe the ion dynamics of [P_666,14_][BEHP] and [P_666,14_][BTMPP] in a supercooled liquid state thoroughly, first, we focus on the shape of the conductivity relaxation process. At first sight in Fig. 2a, the shape of the σ-peak does not change across the broad temperature range. Several dielectric modulus spectra have been superimposed to verify this observation. As presented in Fig. 2c, the time−temperature superposition (TTS) principle is indeed fulfilled for both examined ILs. Thus, the phosphonium ILs with [BEHP]^-^ and [BTMPP]^-^ anions follow the behavior of typical ionic glass-formers^24^ rather than [P_666,14_]-based ILs with the liquid–liquid phase transition (see Fig. 2f). To quantify the shape of the conductivity relaxation process of [P_666,14_][BEHP] and [P_666,14_][BTMPP], the Kohlrausch–Williams–Watts (KWW) function, $\phi (t)=exp[-{(t/{\tau }_{\sigma })}^{{\beta }_{KWW}}]$ has been employed. It is well known that the stretching parameter *β*_KWW_ takes the value between 0 and 1, and it is lower for the broader and more asymmetric peaks^25^. As presented in Fig. 2c, the KWW function with *β*_KWW_ = 0.6 and 0.56 describes the *M*”(*f*) spectra of [P_666,14_][BEHP] and [P_666,14_][BTMPP], respectively. When compared to other [P_666,14_]-based ILs, the examined herein fluids reveal quite broad *M*”(*f*) peaks, i.e., they are characterized by a relatively broad distribution of the relaxation times (see Fig. 2f). It means that in the supercooled state of ILs containing [P_666,14_]^+^ cation and small anions like [SCN]^−^, [TAU]^−^ or [TFSI]^−^, the ions are more dynamically correlated (i.e., components relax with similar τ_σ_), whereas an increase of anion size to [BEHP]^-^ or [BTMPP]^-^ brings higher heterogeneity (i.e., some components are more mobile and some are less mobile).

**Figure 3:**
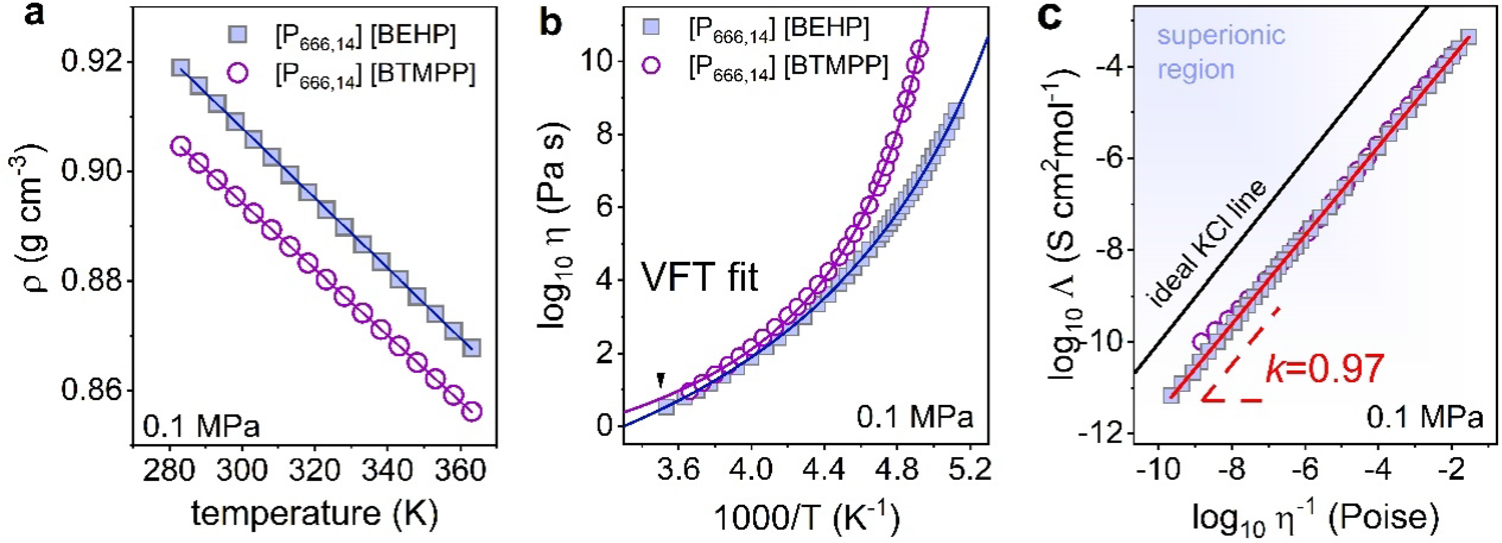
(**a**) Temperature dependence of density for [P_666,14_][BEHP] and [P_666,14_][BTMPP] measured at 0.1 MPa. The solid lines are linear fits. (**b**) Temperature dependence of viscosity for studied ILs. Solid lines denote the fit of the VFT equation. (**c**) Walden plot constructed for studied ILs comparing with ideal KCl line.

The next important aspect of ion dynamics in a supercooled state is the temperature behavior of dc-conductivity *σ*_dc_ and conductivity relaxation times *τ*_σ_. The former is determined directly from the frequency-independent part of *σ*’(*f*) while *τ*_σ_ is calculated as the inverse of *M*’’(*f*) peak maximum (${\tau }_{\sigma }={(2\pi {f}_{\mathrm{max}})}^{-1}$). The obtained results are presented in Fig. 2d,e. At first sight, one can see that the data meet each other at high temperatures, diverge more and more during the cooling and achieve the time scale of 100 s at two temperatures different by 10 K. Thus, both samples reveal the same dc-conductivity at RT conditions; however various *T*_g_. Note that *σ*_dc_(RT) = 1.15∙10^−6 ^Scm^−1^ is around 1.5 decades below the values reported in the past for phosphonium ILs with shorter alkyl tails of a cation^11^. Furthermore, we found that the glass transition temperature defined conventionally as *T*(*τ*_σ_ = 100 s or *σ*_dc_ = 10^−15^ S/cm) quite well agrees with calorimetric *T*_g,_ thereby suggesting that the charge transport is controlled by viscosity in [P_666,14_][BEHP] and [P_666,14_][BTMPP]. The temperature dependence of viscosity has been determined and analyzed in terms of the fractional Walden rule, *Λη*^-*k*^ = const ^26^. to verify this assumption. For this purpose, dc-conductivity needs to be converted to molar conductivity according to the relation, *Λ* = *σ*_dc_*M*_mol_*ρ*^−1^. The temperature evolution of density *ρ* and viscosity *η* obtained experimentally for [P_666,14_][BEHP] and [P_666,14_][BTMPP] are presented in Fig. 3a,b. On the other hand, Fig. 3c shows molar conductivity vs. fluidity on a double logarithmic scale. The solid line represents the ideal Walden behavior assigned to a 0.01 mol dm^−3^ KCl aqueous solution that is a fully dissociated electrolyte with equally mobile ionic species^27,28^. As presented, the fractional exponent *k* is slightly lower than the unity (*k* = 0.97) for both studied ILs. This means that charge transport is indeed strongly coupled to structural dynamics at any *T* in the supercooled liquid state. From Fig. 3c, it is also evident that the experimental data obtained for [P_666,14_][BEHP] and [P_666,14_][BTMPP] fall below the ideal line indicating that, in analogy to other phosphonium ILs^29^, ionization is not complete in these materials.

**Figure 4:**
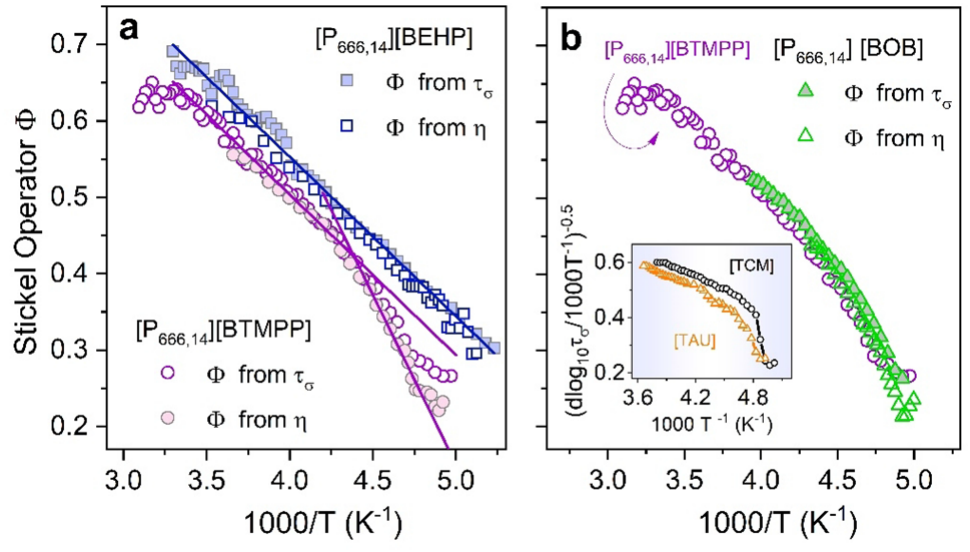
(**a**) The Stickel plots of conductivity relaxation times *τ*_σ_ and viscosity *η* for [P_666,14_][BEHP] and [P_666,14_][BTMPP]. (**b**) The comparison of Stickel analysis for [P_666,14_][BTMPP] with [P_666,14_][BOB]. The inset shows the Stickel analysis of ILs with the LLT phenomenon. Data are taken from ref.^31^.

**Figure 5:**
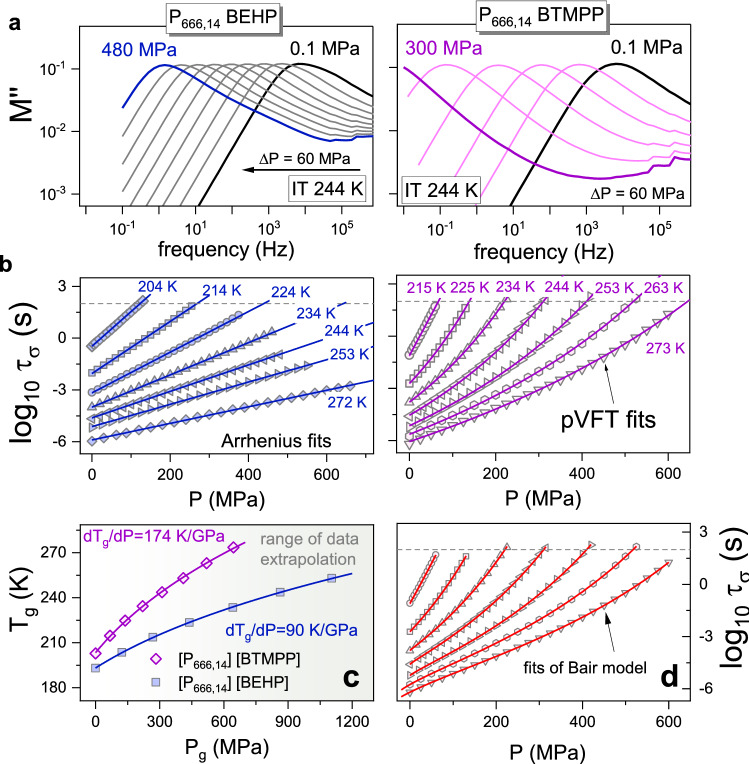
(**a**) The imaginary part of the dielectric loss modulus *M*’’ versus frequency registered during the compression of [P_666,14_][BEHP] and [P_666,14_][BTMPP] at *T* = 244 K. (**b**) Pressure dependence of the conductivity relaxation times *τ*_*σ*_ measured at different isothermal conditions for [P_666,14_][BEHP] and [P_666,14_][BTMPP]. The solid lines denote the corresponding fits, i.e., Arrhenius fit for [P_666,14_][BEHP] and pVTF fit for [P_666,14_][BTMPP]. (**c**) Pressure dependence of the glass transition temperature *T*_*g*_ for studied ILs. The solid lines are fits of the Andersson-Andersson equation to the experimental data. (**d**) Pressure dependence of log_10_ *τ*_*σ*_ (*P*) of [P_666,14_][BTMPP] for the same isotherms. The solid lines represent the fits with the hybrid model (Eq. 4).

From closer inspection of dielectric and viscosity data, one can notice that the Vogel-Fulcher-Tammann equation ${\tau }_{\sigma }(\mathrm{T})={\tau }_{\infty }exp(\frac{D{T}_{0}}{T-{T}_{0}})$ provides proper interpolation of non-Arrhenius *σ*_dc_(*T*^−1^) and *η*(*T*^−1^) dependences of [P_666,14_][BEHP], while for [P_666,14_][BTMPP], the VFT fit deviates slightly from experimental points. For typical glass-forming liquids, such a result designates the existence of the intermediate temperature, *T*_b_, identified with the onset of complex dynamics^30^. Strictly speaking, there are two regions of complex (*T* < *T*_b_) and simple (*T* > *T*_b_) dynamics, characterized by different VFT behavior. Interestingly, considering ILs, only the materials containing imidazolium cation and [TCM] anion revealed a clear *T*_b_ point^31^. To confirm or exclude the presence of *T*_b_ for [P_666,14_][BEHP] and [P_666,14_][BTMPP], the Stickel operator has been employed for dielectric and mechanical data. According to the literature, derivative analysis of temperature variations of the relaxation time (or any other dynamic quantity), $\Phi ={[d(log({\tau }_{\sigma })/d(1000/T)]}^{-1/2}$, transforms single VFT behavior into a linear dependence^32^. On the other hand, when two VFT equations are required to parameterize the experimental data, two linear regions intersect at *T*_b_. The results of the Stickel analysis of *τ*_σ_(*T*^−1^) for studied ILs are presented in Fig. 4a. It clearly shows that [P_666,14_][BTMPP] displays a different behavior than [P_666,14_][BEHP]. Namely, for the latter one, the Stickel plot reveals a single slope while clear deviation from linear behavior is observed for [P_666,14_][BTMPP]. However, in contrast to the Stickel graph of any other glass-forming liquid revealing T_b_^31^, the slope of ${[d(log({\tau }_{\sigma })/d(1000/T)]}^{-1/2}$ dependence is getting larger in the vicinity of the liquid-glass transition; specifically, it grows twice. The analogous analysis performed for viscosity data also confirms such untypical behavior. In this regard, the ion transport in [P_666,14_][BTMPP] follows the behavior of [P_666,14_]-based ILs revealing a liquid–liquid phase transition. The observed effect is not as significant as for [P_666,14_][TCM] or [P_666,14_][TAU], where the LLT was detectable as an abrupt decrease of the Stickel operator, but surprisingly it fully mimics the $\Phi$ trend reported for [P_666,14_][BOB], which undergoes a hidden LLT (see Fig. 4b and inset therein). Thus, one can conclude that in [P_666,14_][BTMPP], LLT occurs in the close vicinity of the liquid-glass transition. At the same time, the first-order phase transition of the same nature does not exist in [P_666,14_][BEHP]. In this context, it is interesting to ask whether these differences bring various pressure sensitivity to examined systems. To explore this issue, a set of isothermal measurements of [P_666,14_][BTMPP] and [P_666,14_][BEHP] has been performed. The representative loss modulus spectra collected at 244 K are shown in Fig. 5a for both examined ILs. We see that isothermal compression has basically the same effect on the ion dynamics as isobaric cooling. Namely, the *M*’’ peak shifts systematically toward lower frequencies with increasing pressure. However, despite the same pressure step, the shift of *M*”(*f*) is much more extensive for [P_666,14_][BTMPP]. Consequently, *M*”(*f*) maximum moves out of the experimental window already at 300 MPa, while for [P_666,14_][BEHP] 480 MPa is not enough. This effect is better visualized when the conductivity relaxation times *τ*_σ_ are plotted vs. pressure. From Fig. 5b, it can be noted that isothermal squeezing makes the conductivity relaxation time longer; however, the *τ*_σ_(*P*) dependences of [P_666,14_][BTMPP] follow the non-Arrhenius behavior, while log*τ*_σ_ grows linearly with increasing pressure for [P_666,14_][BEHP]. The same is observed for *σ*_dc_(T,P) dependences (see Fig. S3 in the supporting information file). Interestingly, the isotherms of [P_666,14_][BTMPP] expose the same performance as high-pressure data of [P_666,14_][BOB]^15^, which again confirms similarities between these two ILs.

The d*T*_g_/d*P* coefficient is usually employed to describe the pressure sensitivity of ion dynamics quantitatively. For this purpose, the liquid glass transition pressure *P*_g_ needs to be determined at various temperature conditions. In analogy to ambient pressure data, we defined *P*_g_ as *P*(*τ*_σ_ = 100 s). Since within the available pressure range, only a few isotherms of [P_666,14_][BEHP] reach the time scale of 100 s, the experimental data have been fitted by using the pressure version of the Arrhenius relation: ${\tau }_{\sigma }={\tau }_{0}\mathrm{exp}\left(\frac{P\Delta V}{RT}\right)$ and then extrapolated to desired conductivity relaxation time. At the same time, to interpolate isotherms of [P_666,14_][BTMPP] pressure counterpart of VFT law (${\tau }_{\sigma }={\tau }_{0}exp(\frac{{D}_{P}P}{{P}_{0}-P})$ has been employed (fits quality will be discussed later). The obtained pressure dependence of liquid-glass transition temperature is presented in Fig. 5c. Due to its non-linear character, the empirical Andersson-Andersson equation: ${T}_{g}={{k}_{1}(1+\frac{{k}_{2}}{{k}_{3}}P)}^{1/{k}_{2}}$ was used to parameterize the data and calculate the d*T*_g_/d*P* coefficient (*k*_1_/*k*_3_)^33^. As expected, markedly different values of d*T*_g_/d*P* (in the limit of ambient pressure) are obtained for [P_666,14_][BTMPP] and [P_666,14_][BEHP]; that is 174 K/GPa and 90 K/GPa, respectively. Interestingly, d*T*_g_/d*P* of [P_666,14_][BOB] is between these two limits. The obtained results suggest that in [P_666,14_][BEHP], there are strong van der Waals interactions between alkyl chains of cations and anions. Such a constituted structure becomes hard to break and thus is weakly sensitive to compression. The same is impossible for [P_666,14_][BTMPP] due to the branched 5-carbon chains of the anion. Consequently, squeezing seems to strongly reduce the free volume between the alkyl chains and brings significant pressure sensitivity of *T*_g_.

**Figure 6:**
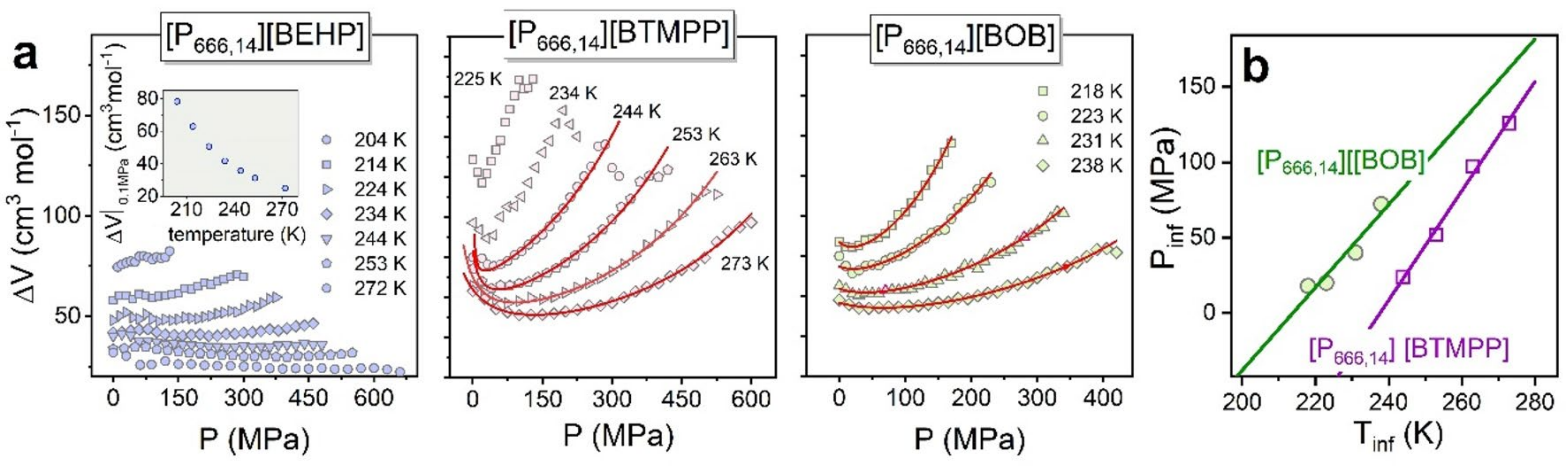
(**a**) Pressure dependences of activation volume for [P_666,14_][BEHP] [P_666,14_][BTMPP] and [P_666,14_][BOB]. The lines are fits according to Eq. (4). In the inset, the temperature dependence of activation volume of [P_666,14_][BEHP] at ambient pressure is shown. (**b**) Temperature dependence of inflection pressure for [P_666,14_][BTMPP] and [P_666,14_][BOB]. Solid lines are linear fits, extrapolating to the inflection temperature at 0.1 MPa.

Coming back to the discussion on *τ*_σ_(*T,P*) dependences, one can notice that volume-activated law quite well parameterizes the data of [P_666,14_][BEHP]. However, pVFT deviates from experimental points, especially at high temperatures and low pressures. Therefore, in the next step, we employed Avramov entropic model^34^ for data analysis. The mathematical expression of this model is

3 $$log\tau_{\sigma } \left( {T,P} \right) = log\tau_{\infty } + \left( {\frac{{T_{r} }}{T}} \right)^{{\alpha_{0} \left[ {1 - k\ln \left( {1 + \frac{P}{\Pi }} \right)} \right]}} \left( {1 + \frac{P}{\Pi }} \right)^{\beta } log\frac{{\tau_{g} }}{{\tau_{\infty } }}$$

where the pre-exponential factor *τ*_∞_ denotes the conductivity relaxation times at high temperatures, *T*_r_ is a reference temperature (e.g., *T*_g_), log*τ*_g_ = 2 since we previously defined *T*_g_ as the temperature at which *τ* = 100 s, and *Π, k, α*_0_, and *β* are fitting parameters. A significant advantage of this model is that it accounts for isothermal and isobaric conductivity relaxation times simultaneously. Although it was introduced to predict the viscosity of glass-formers at various temperature and pressure conditions, the Avramov model has been satisfactorily applied to describe the ion dynamics of aprotic ionic liquids, where viscosity controls ion transport^35^. As displayed in Fig. S4, the isothermal and isobaric dielectric relaxation data form a two-dimensional plot. The wire surface, in turn, represents the result of global fitting analysis using Eq. (3). It is clearly visible that the generated surface shows good agreement with the experimental *τ*_σ_(*T*,*P*) data for both studied ILs. However, when the fitting curves have been transferred to conventional representation (see Fig. S4b), a poor agreement between fits and the experimental points is again visible for [P_666,14_][BTMPP] at high temperatures. Therefore, the derivative analysis has been performed to look closer at *τ*_σ_(*T*,*P*) dependences. Precisely, the apparent activation volume $\Delta V=2.303RT{(\frac{\partial {log}_{10}{\tau }_{\sigma }}{\partial P})}_{T}$ that provides information on the volume required for local molecular motion has been calculated. Note that for linear *τ*_σ_(*P*) dependences, constant Δ*V* is expected, while for non-Arrhenius log*τ*_σ_(*P*), it continuously increases with pressure. Δ*V*(*P*) determined at various T conditions for [P_666,14_][BEHP] and [P_666,14_][BTMPP] are depicted in Fig. 6a. Keeping the same scale of Y-axis, it becomes obvious that *ΔV* of [P_666,14_][BEHP] is markedly lower and weakly dependent on pressure, especially at high temperatures. Furthermore, it decreases with increasing temperature, which is typical for glass-forming liquids. Compared to activation volume determined for imidazolium-based ionic liquids, it is markedly larger^36^, which reflects the big size of relaxing ions. On the other hand, unusual behavior of Δ*V*(*P*) is observed for [P_666,14_][BTMPP]. In particular, the activation volume decreases upon compression at the lower pressure range and increases with pressure in the higher pressure region. Consequently, Δ*V*(*P*) reveals a clear minimum called an inflection point that indicates the concave-convex character of log*τ*_σ_(*P*) dependences.

In 1993, Herbst et al.^37^ proposed that this inflection point might arise from a non-linearity of the volume with a change of pressure. On the other hand, Casalini and Bair^38^ suggested that it should be attributed to the pressure dependences of the compressibility and the apparent activation energy at constant volume. In order to analyze this phenomenon in more detail, a phenomenological model proposed by Bair^39^, employed initially to describe inflections in pressure dependence of viscosity, can be used here to parametrize the pressure dependence of conductivity relaxation times. This model combines the pVFT equation with the McEwen equation ($\eta (P)={\eta }_{0}{(1+\frac{{\alpha }_{0}}{q}P)}^{q}$), and can be appropriate for low and high-pressure ranges, respectively,

4 $$\tau_{\sigma } = \tau_{0} \left( {1 + \frac{{\alpha_{0} }}{q}P} \right)^{q} exp\left( {\frac{{D_{P} P}}{{P_{0} - P}}} \right)$$

Here, *q* and *α*_0_ are the McEwen exponent and parameter, respectively. The fits of this hybrid model to *τ*_σ_(*P*) isothermal data of [P_666,14_][BTMPP] are presented in Fig. 5d. As can be seen, the fitting curves follow the experimental points perfectly and consequently correctly interpolate the Δ*V*(*P*) dependences of [P_666,14_][BTMPP]. From Fig. 6b, one can note that the inflection point is observed at higher pressures as the temperature increases, and a linear dependence describes well the obtained data. Namely, the inflection pressure increases with temperature at a slope of 3.6 MPa/K. Extrapolation of this linear fit to ambient pressure gives *T*_infl_(*P* = 0.1 MPa) equal to 238 K, which is above the dynamic glass transition temperature.

Interestingly, so far, only a few glass formers have been compressed strongly enough to observe the inflection point experimentally^40^. Among them, one can mention protic ionic liquid [C_8_Him][NTf_2_]^41^, glycerol^42^ and 2-butyl-1-octanol. Interestingly, all of them can form supermolecular structures due to the strong H-bonding network. In this context, one could expect that [P_666,14_][BTMPP], as well as [P_666,14_][BOB], reveal some ordering tendencies that affect their ion dynamics at ambient and elevated pressure and finally become the origin of the LLT.

## Conclusions

In summary, we examined the dielectric properties of trihexyltetradecylphosphonium bis(2-ethylhexyl) phosphate ([P_666,14_][BEHP]) and trihexyltetradecylphosphonium bis(2,4,4-trimethylpentyl) phosphinate ([P_666,14_][BTMPP]) over a wide temperature and pressure range. These materials can be classified as good glass-forming liquids, as confirmed by DSC scans. However, at the same time, there is no clear calorimetric evidence of the liquid–liquid phase transition being observed previously for other [P_666,14_]-based ILs. Furthermore, the dynamics of examined ILs shows the features typical for ionic conductors, that is non-exponential behavior of conductivity relaxation peak *M*”(*f*) and non-Arrhenius temperature dependence of *τ*_σ_(*T*^−1^). Although the dielectric parameters (i.e., *τ*_σ_, *σ*_dc_, and *β*_KWW_) did not reveal any signs of LLT, a detailed analysis of *τ*_σ_(*T*^−1^) and *η*(*T*^−1^) dependences revealed an unusual curvature of Stickel plot for [P_666,14_][BTMPP]. Such a peculiar behavior of ion dynamics near T_g_ has not been observed before for any other phosphonium IL, instead of [P_666,14_][BOB], for which the LLT was postulated to overlap the liquid-glass transition. Thus, one can conclude that LLT and T_g_ occur in close vicinity for IL with [BTMPP]^-^ anion. At the same time, an increase in the Stickel slope at *T*_b_ can be identified as the fingerprint of changes in ion dynamics accompanying LLT. The high-pressure dielectric measurements revealed that the ion dynamics of [P_666,14_][BTMPP] is much more similar to [P_666,14_][BOB] than to [P_666,14_][BEHP]. In particular, *τ*_σ_(*P*) of the latter follows the volume-activated law, while non-Arrhenius behavior characterizes the isotherms of the first two ILs. Specifically, the concave-convex character of log*τ*_σ_(*P*) dependences has been observed for [P_666,14_][BTMPP] and [P_666,14_][BOB]. Consequently, a unique phenomenon called an inflection point was found to characterize the high-pressure dynamics of ionic systems with hidden LLT.

## Method

### Materials

Investigated ionic liquids trihexyltetradecylphosphonium bis(2-ethylhexyl) phosphate ([P_666,14_][BEHP], M_W_ = 805.27 gmol^−1^) and trihexyltetradecylphosphonium bis(2,4,4-trimethylpentyl) phosphinate ([P_666,14_][BTMPP], M_W_ = 773.27 gmol^−1^) were purchased from io-li-tec Ionic Liquids Technologies as transparent liquids (([P_666,14_][BTMPP]-bright yellow, [P_666,14_][BEHP]-colorless). The purity tests, including NMR, have been made, and they agree with those provided by Iolitec. The bright yellow color can indicate the halide impurities in [P_666,14_][BTMPP]. Before the experiments, samples were dried overnight in a Vacutherm oven (1000 mbar, 12 h, 70 °C). The color of the samples did not change after drying. The water content of both ILs is less than 0.1%, determined by Metrohm 899 coulometer.

### Differential scanning calorimetry measurements

Thermodynamic properties of studied ILs were examined using a Mettler-Toledo DSC instrument equipped with a HSS8 ceramic sensor with 120 thermocouples and a liquid nitrogen cooling accessory. The calibrations for temperature and enthalpy were performed by using indium and zinc standards. The samples (approx. 20 mg) were sealed in aluminum crucibles (40 μL) with pierced lid. Each IL was measured from 123.15 to 303.15 K with a 10 K min^−1^ heating rate. A new sample was prepared for the same temperature cycles three times to ensure reproducibility and high accuracy.

### Dielectric measurements

We have used a glove box to prepare samples for dielectric measurements. The dielectric measurements in a wide frequency range of 10^−2^–10^7^ Hz were performed at ambient pressure using a Novo-Control GMBH Alpha dielectric spectrometer. The same stainless steel electrodes (diameter = 15 mm) with a fixed distance (0.1 mm) provided by the quartz ring were used for both studied ILs. During the measurements, the temperature was controlled by a Quatro system using a nitrogen gas cryostat with an accuracy of 0.1 K. The Karl Fisher test made after the dielectric experiments did not show moisture uptake. The dielectric measurements at elevated pressure were carried out by means of a high-pressure system developed by Unipress over a frequency range from 10^−2^ to 10^6^ Hz with a specially designed capacitor. The capacitor was filled with the studied sample and sealed by a Teflon capsule during the measurements. Then the whole part was placed in the high-pressure chamber and compressed using silicone oil. The pressure was controlled with a resolution of 1 MPa by means of the Unipress setup, and a Weiss fridge adjusted the temperature within 0.1 K.

### Viscosity measurements

The viscosity was measured employing an ARES G2 Rheometer. In the supercooled liquid region, aluminum parallel plates of diameter 4 mm were used. The rheological experiments were performed in the frequency range from 0.1 to 100 rad s^−1^ (10 points per decade) with strain equal to 0.01% in the vicinity of the liquid glass transition. The strain was increased by one order of magnitude with every 10 K. The relative uncertainty of the reported viscosity measurements *u*_r_(*η*) from calibration, temperature and pressure control did not exceed 7%.

### Density measurements

Density was measured in the temperature range from 283.15 to 363.15 K using a vibrating-tube densimeter DMA 4500 M (Anton Paar, Austria). The apparatus was calibrated directly before measurements with dry air and bi-distilled water. The water was freshly degassed (by boiling) before use. The viscosity-related errors were automatically corrected in full range.

## Supplementary Information

Supplementary Figures.

## Data Availability

All data generated or analyzed during this study are included in this published article (and its Supplementary Information files). Source data are provided in this paper.
