# Supplementary material for: Effect of bulky anions on the liquid-liquid phase transition in phosphonium ionic liquids: Ambient and high-pressure dielectric studies
Source: Sci Rep. 2023 Feb 21;13:3040. doi: 10.1038/s41598-023-29518-8 (PMC9944924; doi:10.1038/s41598-023-29518-8)
Supplement: Supplementary file 1 — Supplementary Figures. [file 41598_2023_29518_MOESM1_ESM.docx]

*Supporting information*

**Effect of bulky anions on the liquid-liquid phase transition in phosphonium ionic liquids: Ambient and high-pressure dielectric studies**

B. Yao^1^, M. Paluch^1^, Z. Wojnarowska^1*^

*^1^Institute of Physics, University of Silesia, SMCEBI, 75 Pulku Piechoty 1A, 41-500 Chorzow, Poland*

^*^corresponding author: zaneta.wojnarowska@us.edu.pl

**Supplementary Figures**

**Figure S1** The temperature dependence of heat capacity on different cooling and heating rate: 10 (black), 5 (blue), 2 (green), 1 (magenta) K/min for [P_666,14_][BTMPP] a and [P_666,14_][BEHP] b. Inset: Comparison between DSC scans obtained during the standard heating rate 10 K/min (navy) and after 6 hours-aging process performed in the glassy state (orange) at 193 K for [P_666,14_][BTMPP] and at 173 K for [P_666,14_][BEHP].

Generally, the secondary relaxations in glass formers can be classified depending on how strongly they are correlated to the primary relaxation. Namely, secondary relaxations are identified as the Johari-Goldstein (JG) relaxation, which involves a certain (intermolecular) motion of the entire molecule, and non-JG relaxation, which involves a part of the molecule (intramolecular) motion. Hence, JG relaxation is considered an indispensable precursor for cooperative structure relaxation and is characterized by sensitivity to density changes. Non-JG relaxations, by contrast, originate from intramolecular local motion, are pressure insensitive and have lower activation energy. To fast-check the origin of the secondary mode, the coupling model (CM) can be used:

$\tau_{JG}\approx t_{c}^{1-\beta_{KWW}}\tau_{\alpha}^{\beta_{KWW}}$ (1)

where *t_c_* = 2 ps is a temperature-independent constant, *β_KWW_* is the stretching parameter. It has been confirmed that the CM is still valid by replacing *τ_α_* with *τ_σ_* in Eq. (1) for ionic glass formers. We can see from Fig S2, that the determined values of *τ_JG_* for [P_666,14_][BTMPP] and [P_666,14_][BEHP] do not agree with the relaxation times of the *β*-process.

**Figure S2** The comparison of the temperature dependence of *τ_β_* and *τ_JG_* for [P_666,14_][BEHP] and [P_666,14_][BTMPP].

**Figure S3** The dc-conductivity as a function of pressure for [P_666,14_][BEHP] and [P_666,14_][BTMPP] at several isothermal conditions.


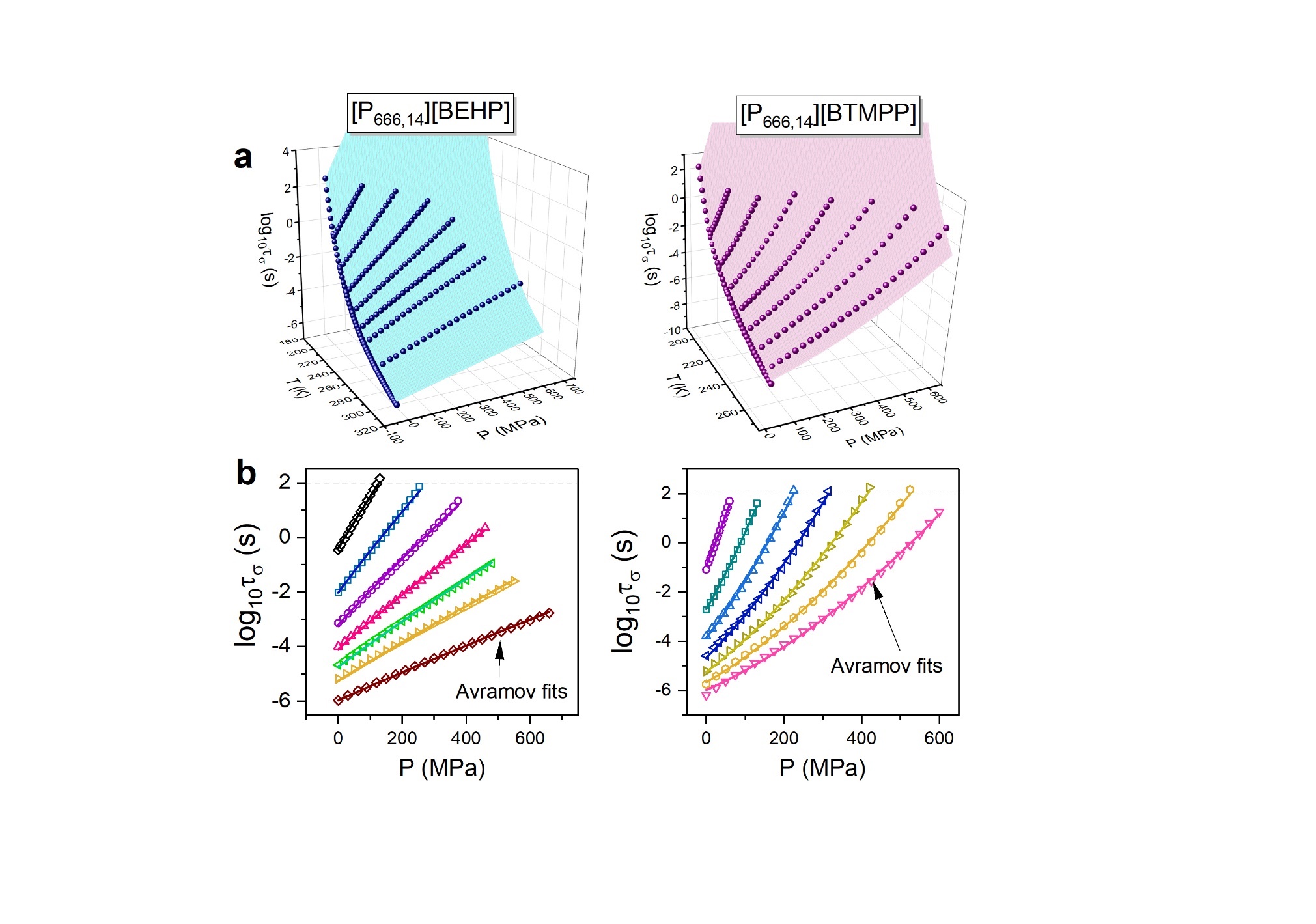


**Figure S4** **a** The conductivity relaxation times are plotted as a function of both temperature and pressure in a 3D plane for studied ILs. The surface is determined by fitting the experimental data to the Avramov model with the following parameters: 1) log_10_(τ_∞_)=-7.55±0.03, k=0.101±0.004, α_0_=5.26±0.03, β=0.72±0.02, and Π=271.8±10.9 MPa for [P_666,14_][BEHP] with T_r_=T_g_=193.0 K; 2) log_10_(τ_∞_)=-7.07±0.06, k=-0.019±0.014, α_0_=7.20±0.11, β=1.68±0.07, and Π=267.42±14.87 MPa for [P_666,14_][BTMPP] with T_r_=T_g_=202.9 K. **b** Test of the Avramov model for pressure dependence of conductivity relaxation times for both samples. Solid lines indicate fits of the Avramov model.
